# Association between continuity of care and type 2 diabetes development among patients with thyroid disorder

**DOI:** 10.1097/MD.0000000000018537

**Published:** 2019-12-27

**Authors:** Sang Ah Lee, Dong-Woo Choi, Junhyun Kwon, Doo Woong Lee, Eun-Cheol Park

**Affiliations:** a Department of Public Health, Graduate School; b Institute of Health Services Research, Yonsei University, Seoul; c Research and Analysis Team, National Health Insurance Service Ilsan Hospital, Goyang; d Department of Preventive Medicine, Yonsei University College of Medicine, Seoul, Republic of Korea.

**Keywords:** continuity of care, diabetes, hyperthyroidism, hypothyroidism, thyroid dysfunction

## Abstract

Thyroid disorders are associated with blood glucose abnormalities. For rendering the patients euthyroid, routine screening and care are essential. Therefore, the aim of this study was to investigate the association between continuity of care (COC) and type 2 diabetes onset among patients with thyroid disorders.

We used the national claim data. Our study population was 4099 patients with hyperthyroidism or hypothyroidism. For calculating COC, the Most Frequent Provider Continuity Index (MFPCI), Modified Modified Continuity Index (MMCI), and COC Index (COCI) were used. The dependent variable was type 2 diabetes onset. The Cox proportional hazard regression model was used.

Among 4099 patients with thyroid disorders, 25.3% experienced onset of type 2 diabetes. Thyroid patients who had MFPCI and COCI below the median were more likely to experience onset of type 2 diabetes than who had these indices above the median (MFPCI: hazard ratio [HR] = 1.26, 95% confidence interval [CI] = 1.09–1.46; COCI: HR = 1.22, 95% CI = 1.06–1.41). Our subgroup analysis showed that female patients and those 20 to 34 years of age showed a significant association between COC and onset of type 2 diabetes.

Patients with thyroid disorders with low COC showed an increased risk of developing type 2 diabetes. Therefore, efforts to enhance COC among patients with thyroid disorders needs to be encouraged.

## 1 Introduction

Thyroid dysfunction is one of the most prevalent endocrine disorders.^[1]^ According to the American Association of Clinical Endocrinologists, an estimated 13 million people, representing 4.8% of the population of the United States, have undiagnosed thyroid dysfunction.^[2]^ The prevalence of thyroid dysfunction is on the rise in South Korea. According to the Health Insurance Review and Assessment Service, the number of people with thyroid dysfunction has increased from 914,000 in 2009 to 1,264,000 in 2016.^[3]^ Along with this trend, comorbidities including heart failure,^[4]^ atrial fibrillation,^[5]^ cardiovascular diseases,^[6]^ depression,^[7]^ and metabolic disorders^[8]^ have also increased. Previous studies have focused on the relationship between thyroid dysfunction and diabetes.[^9^,^10^,^11^,^12^,^13^]

Thyroid dysfunction and diabetes are the two most common endocrine system disorders. Furthermore, these two diseases are closely related,[^9^,^10^,^11^,^12^,^13^] as evidenced by the term “thyroid diabetes.”[^9^,^10^,^11^,^12^,^13^,^14^,^15^] Previous studies have studied the relationship between thyroid hormone and blood glucose. According to these studies, hyperthyroidism is related to hyperglycemia as a result of increased glucose gut absorption owing to the excess thyroid hormones.^[13]^ In addition, increased proinsulin levels are observed in untreated Graves’ disease.^[12]^ Hypothyroidism can also be related to decreased glucose intolerance and insulin sensitivity.[^10^,^16^] Moreover, it has been reported that hypothyroidism is associated with metabolic syndrome and could be indirectly related to the diabetes onset.^[17]^ Insulin and glucose status may also be unstable owing to the effect of abnormal thyroid hormone levels.[^11^,^12^,^13^] Therefore, controlling thyroid hormones is imperative to prevent the onset of diabetes among patients with thyroid dysfunction.

According to the previous study, appropriate timely treatment along with routine screening programs could prevent negative outcomes.^[1]^ In this respect, continuity of care (COC) could be one of the appropriate methods to control a patient's thyroid hormone status and monitor the onset of complications. COC means a continuous relationship between physicians and patients.^[18]^ A high level of COC has been associated with positive outcomes including better quality of care,^[19]^ treatment adherence,^[20]^ and improved self-management.^[21]^ Particularly, this concept is important for patients with chronic disease because it can prevent sudden aggravation of the condition.^[22]^

Although COC is an important concept for patients with chronic diseases,^[23]^ there are few studies on the outcome of COC in patients with thyroid dysfunction. Therefore, this study aimed to investigate the association between COC and onset of type 2 diabetes (T2D) among patients with thyroid dysfunction in South Korea using nationwide claim data. We hypothesized that patients with thyroid dysfunction along with low COC are more likely to experience onset of T2D.

## 2 Methods

### 2.1 Data and study population

We used the Korean National Health Insurance claims database from 2002 to 2013, which includes the claims data of 1,025,340 individuals, accounting for 2% of the South Korean population. The cohort population was selected using random sampling methods. This data is secondary data and does not contain any data which can identify individual. Therefore, ethical approval is exempted. The requirement for informed consent was waived because this study was based on routinely collected claims data.

Our study population included those who visited medical institutions with thyroid disorders (International Classification of Disease [ICD] codes E01–03, hypothyroidism, and E05, hyperthyroidism). To calculate COC, we excluded those who visited medical institutions fewer than four times during 2 years from the onset of their thyroid disorder diagnosis. Additionally, we also excluded those whose onset of thyroid disorder year was in 2012 or 2013, in consideration of the follow-up time. Therefore, the final study population included 4099 patients with thyroid disorders.

### 2.2 Variables

We used three indices for measuring COC. First, the Most Frequent Provider Continuity Index (MFPCI) measures the frequency of visits to the most frequent provider. Second, the Modified Modified Continuity Index (MMCI) measures not only the total number of visits to medical institutions but also the total number of providers available. This index reflects the number and distribution of visits to other providers. Third, the COC Index (COCI) integrates both the MFPCI and MMCI. The COCI measures an individual's COC while considering the effect of the total number of providers and total number of visits. 

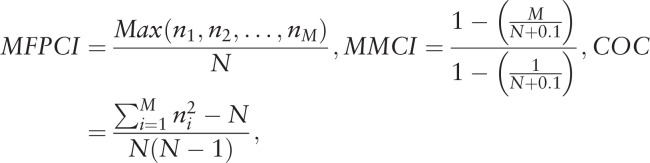



where N is the total number of visits, *n*_*i*_ is the number of visits to provider *i*, *M* is the total number of providers.

Our dependent variable is T2D onset. Patients who visited a medical institution and were diagnosed with T2D (ICD-10 code E11) after 2 years from the onset of a thyroid disorder were classified into the T2D onset group. Patients with no record of T2D were classified into the non-onset group.

We controlled for age, sex, income, insurance type, residential area, type of thyroid disorder, Charlson Comorbidity Index, presence of a disability, type and location of main attending clinic, and thyroid disorder onset year. Age was classified into four groups (20–34, 35–54, 55–64, and ≥65 years). As the data do not contain exact income, health insurance premiums were used to estimate income based on three categories (low; the bottom 20% of premiums; middle; 20% to 80% of premiums, high; the top 20% of premiums). Residential area was classified into capital area, metropolitan area, and rural area. The type of thyroid disorder was classified as hypothyroidism (ICD-10 codes E01-E03) or hyperthyroidism (ICD-10 code E05). The type of main attending clinic was classified as general hospital, hospital, or clinic. The location of the main attending clinic was categorized the same as the residential area.

### 2.3 Statistical analysis

The chi-squared test and Student's *t* test were used to examine the general characteristics of the study population. To investigate the association between COC and onset of T2D among patients with thyroid disorders, Cox proportional hazards model was used to calculate hazard ratios (HRs) by adjusting for control variables. We also conducted a set of subgroup analyses stratified by age, sex, income, and type of thyroid disorder. All statistical analyses were conducted using SAS software (version 9.4; SAS, Cary, NC). Statistical significance was set at *P* < .05.

## 3 Results

Table 1 shows the general characteristics of the study population. Among 4099 patients with thyroid disorders, 1036 (25.3%) experienced an onset of T2D. The mean MFPCI, MMCI, and COCI were 0.897 ± 0.165, 0.940 ± 0.105, and 0.842 ± 0.235, respectively. Among those whose MFPCI, MMCI, and COCI were less than the median, 29.3% (n = 250), 27.3% (n = 330), and 28.9% (n = 330) experienced T2D, respectively.

**Table 1:**
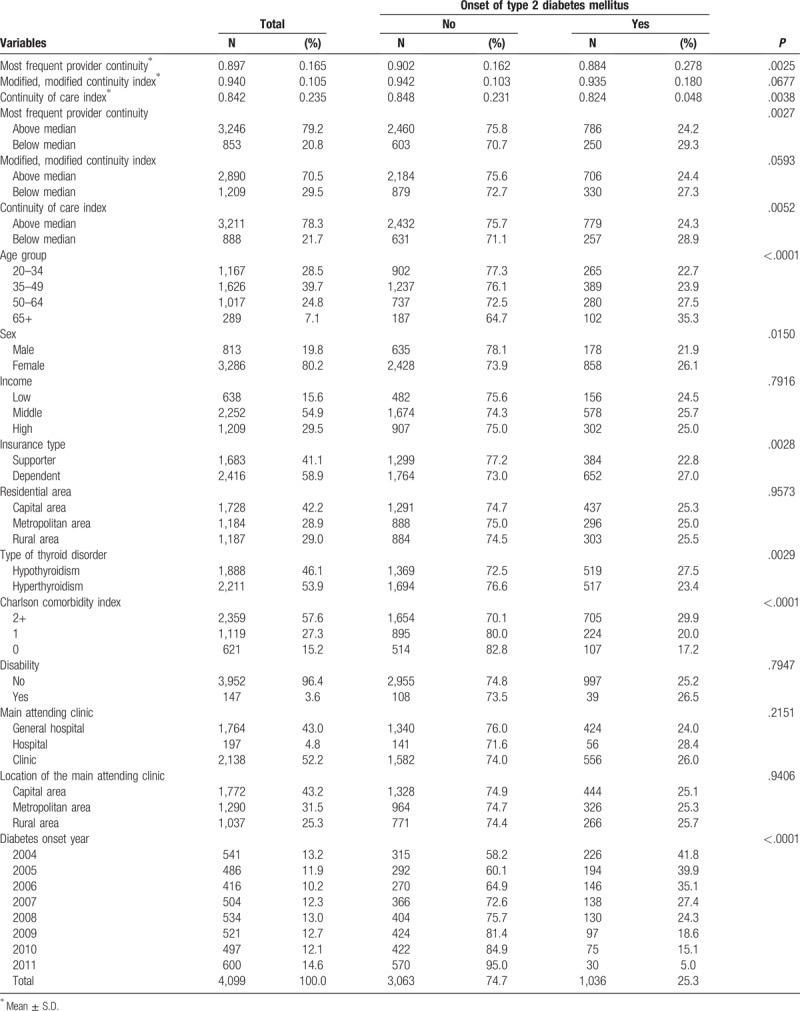
General characteristics of the study population.

Table 2 shows the association between COC indices and onset of T2D. Thyroid patients with their MFPCI and COCI less than the median were more likely to experience an onset of T2D than were those with values exceeding the median (MFPCI: HR = 1.26, 95% CI = 1.09–1.46; COCI: HR = 1.22, 95% CI = 1.06–1.41). MMCI did not show a statistically significant association; however, those with values below the median had a higher HR (HR = 1.14, 95% CI = 1.00–1.30), the same as the other indices.

**Table 2:**
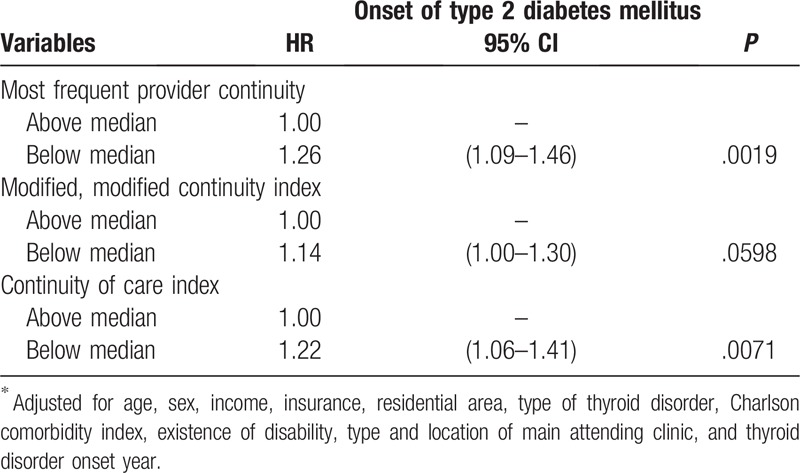
Association between continuity of care indices and onset of type 2 diabetes^∗^.

Table 3 shows the results of a set of subgroup analyses stratified by type of thyroid disorder, income, age group, and sex. Both hypothyroidism (HR = 1.31, *P* = .0160) and hyperthyroidism (HR = 1.23, *P* = .0405) showed a significant association with the onset of T2D when the MFPCI was below the median. Only hypothyroidism showed a statistically significant association with onset of T2D when the COCI was below the median (HR = 1.27, *P* = .0284). Regarding income, the higher income group did not show any association of COC and onset of T2D. Regarding age group, only the 20 to 34 age group showed an association with onset of T2D for MFPCI (HR = 1.34, *P* = .0237), MMCI (HR = 1.30, *P* = .0342), and COCI (HR = 1.31, *P* = .0330) values below the median. Considering sex, female patients showed a significant association with diabetes onset when their COCI was below the median COC, whereas male patients did not show any association.

**Table 3:**
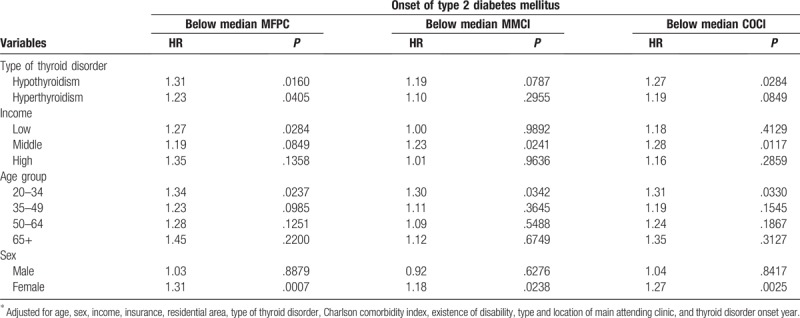
Association between continuity of care indices and onset of type 2 diabetes stratified by type of thyroid disorder, income, age, and sex^∗^.

## 4 Discussion

In this study, we aimed to examine the association between diverse COC indices including MFPCI, MMCI, and COCI and onset of T2D among patients with thyroid dysfunction. The result of this study is in line with the results of previous studies that evaluated the effect of COC on health outcomes.[^24^,^25^,^26^] While limited studies have previously evaluated the effect of COC in patients with thyroid dysfunction, our study showed that patients having thyroid dysfunction with low COC were more likely to experience onset of T2D, and this association was generally maintained for all three indices (MFPCI, MMCI, and COCI). Regarding subgroup analysis, this association was also observed regardless of the type of thyroid dysfunction (hyperthyroidism or hypothyroidism). Additionally, female patients and patients aged 20 to 34 years showed a significant association between COC and onset of T2D.

The association between abnormal thyroid hormone levels and diabetes has been studied for several decades, and many studies have shown such an association.[^15^,^27^,^28^,^29^] A point of similarity is that abnormal thyroid hormone levels increased the risk of developing diabetes.[^12^,^13^] Hyperthyroidism has been related to increase in liver gluconeogenesis, increased peripheral insulin resistance, and it showed an association with glucose intolerance.[^30^,^31^] Hypothyroidism has also been found to be associated with decreased glucose intolerance and peripheral insulin sensitivity,[^16^,^32^] and improved insulin sensitivity was observed with treatment of hypothyroidism.^[33]^ Furthermore, a previous study reported that hypothyroidism was associated with metabolic syndrome and could be related to diabetes onset, indirectly.^[17]^ Therefore, controlling thyroid hormones levels within the normal range is imperative in patients with thyroid dysfunction to prevent the development of diabetes.^[28]^

For treating hyperthyroidism, the production and secretion of stimulant-autoantibody about the TSH receptor should be suppressed. Thus, anti-thyroid medications that suppress the production and secretion of thyroid hormone are used to maintain normal thyroid function. According to the diagnosis and treatment guideline of thyroid disorders,[^34^,^35^] 97.1% of endocrinologists in Korea use medications (e.g., antihyperthyroid medication) as the initial treatment. The main reason of treatment failure is non-adherence to medication,^[36]^ and thus, regular follow-up is important. The guideline recommends conducting follow-up 4 weeks after the first intake of medication, with 4 to 8 weeks of follow-up until normal state is achieved. The response to treatment is assessed based on free T4 and T3 or TSH levels. A similar treatment process is followed for hypothyroidism as well. For patients with hypothyroidism, supplement of thyroid hormone is needed. Intake of thyroid hormone supplement is similar to normal thyroid hormone secretion and involves similar mechanisms, in which thyroid hormone is secreted as T4 and converted to T3. Therefore, maintaining TSH or FT4 in the normal range is the target of treatment. Hypothyroidism requires follow-up 4 to 6 weeks after taking thyroid hormone, with continuous follow-up for about 6 to 12 months. Thus, COC is important in the follow-up period. If patients go to another physician without their medical records, the treatment process could be mixed up. Therefore, low COC could influence thyroid function. In summary, low COC makes it difficult to follow up the change in thyroid function according to the treatment. In this process, considering its well-known benefits, COC could contribute to normalizing thyroid hormone levels.

COC is related to better outcomes of care owing to several positive effects. From the perspective of physicians, they can obtain better knowledge about their patients and detect possible problems sooner[^37^,^38^] because they can provide treatment continuously and do not have to check their status from the beginning. In terms of patients, those with high COC have high trust in their physicians, which is potentially related to their medication or treatment compliance.^[39]^ Furthermore, patients with high COC have a tendency to show better self-management behaviors based on this trust.^[21]^

Considering that an unstable thyroid hormone status could impair glucose control,^[27]^ maintaining consistent treatment is necessary to stabilize the thyroid hormones. Maintaining stable levels of thyroid hormones may help in avoiding unstable blood glucose control. According to the treatment guidelines for patients with thyroid disorders, periodic monitoring is imperative for judging the response to treatment and controlling the dosage of medication according to the patient's status.^[40]^ In addition, monitoring a newly diagnosed patient's status regularly until their status becomes stable is recommended.^[40]^ Therefore, the concept of COC is needed. By regularly following up the thyroid hormone status and administering proper treatment for the patient's condition, thyroid hormones can be well managed.

Our subgroup analysis showed that patients in the low- or middle-income group and the young adult group as well as female patients showed an increased risk of developing diabetes when their COC indices were low. In the high-income group, there was no statistically significant association. Generally, low socioeconomic groups lack social networks that provide much information about disease.^[41]^ Therefore, visiting physicians regularly is important to obtain knowledge about management of their disease or other information. Young adults have a relatively low risk of developing diabetes, and diabetes often occurs in middle-aged or older individuals.^[42]^ However, in this study, patients with thyroid disorders in the 20 to 34 age group showed a higher rate of diabetes when they had a low COCI. Therefore, COC should be carefully considered especially in young adults.

The results of this study should be interpreted carefully owing to several limitations. First, this study used claims data, and the accuracy of such data has been controversial for several years because the original purpose of these data was for claiming instead of research. However, a study on the accuracy of these data showed almost 70% accuracy.^[43]^ Second, these data did not contain information about the severity of the diabetes and thyroid disorders. Furthermore, the level of thyroid hormone or blood glucose cannot be investigated because the data did not contain lab information. In addition, other factors such as personal lifestyle behaviors such as smoking, drinking, or physical activities, which could affect the development of diabetes were not available. This lack of information could generate a potential confounding bias. Third, although we washed out the first year of the cohort study by extracting newly diagnosed patients with thyroid dysfunction, there could be contamination because there were no data before 2002. Fourth, the physicians who provided medical services could not be identified. Thus, we calculated COC based on medical institutions. However, a strength of this study is the investigation of the association between thyroid disorders and diabetes by adopting the COC concept.

## 5 Conclusion

Considering the increasing prevalence of thyroid disorders in Korea, additional attention must be paid to the problems resulting from thyroid disorders. Considering the relationship between thyroid disorders and diabetes, an effort to minimize the effect of thyroid disorders on the development of T2D is needed. According to the results of this study, which found that patients with thyroid disorders with low COC showed an increased risk of developing T2D, efforts to enhance COC for patients with thyroid disorders should be encouraged. Further study should be conducted by using laboratory data, and the differences in thyroid hormone levels according to COC levels should be investigated.

## Author contributions

**Conceptualization:** Sang Ah Lee.

**Formal analysis:** Dong-Woo Choi.

**Methodology:** Junhyun Kwon.

**Supervision:** Eun-Cheol Park.

**Writing – original draft:** Sang Ah Lee.

**Writing – review & editing:** Doo Woong Lee.

Eun-Cheol Park: 0000-0002-2306-5398.

Eun-Cheol Park orcid: 0000-0002-2306-5398.

## References

[1] Garmendia Madariaga A Santos Palacios S Guillén-Grima F The incidence and prevalence of thyroid dysfunction in Europe: a meta-analysis. J Clin Endocrinol Metab 2014;99:923–31. 24423323 10.1210/jc.2013-2409

[2] Garber JR Cobin RH Gharib H Clinical practice guidelines for hypothyroidism in adults: cosponsored by the American Association of Clinical Endocrinologists and the American Thyroid Association. Thyroid 2012;22:1200–35. 22954017 10.1089/thy.2012.0205

[3] Health Insurance Review & Assessment Service, National Health Insurance Service. *National Health Insurance Statistical Yearbook.* Wonju: Health Insurance Review & Assessment Service, National Health Insurance Service; 2017.

[4] Rodondi N Bauer DC Cappola AR Subclinical thyroid dysfunction, cardiac function, and the risk of heart failure: the Cardiovascular Health Study. J Am Coll Cardiol 2008;52:1152–9. 18804743 10.1016/j.jacc.2008.07.009 PMC2874755

[5] Selmer C Olesen JB Hansen ML The spectrum of thyroid disease and risk of new onset atrial fibrillation: a large population cohort study. BMJ 2012;345:e7895. 23186910 10.1136/bmj.e7895 PMC3508199

[6] Cappola AR Fried LP Arnold AM Thyroid status, cardiovascular risk, and mortality in older adults. JAMA 2006;295:1033–41. 16507804 10.1001/jama.295.9.1033 PMC1387822

[7] Ittermann T Völzke H Baumeister SE Diagnosed thyroid disorders are associated with depression and anxiety. Soc Psychiatry Psychiatric Epidemiol 2015;50:1417–25. 10.1007/s00127-015-1043-0 25777685

[8] Mullur R Liu Y-Y Brent GA Thyroid hormone regulation of metabolism. Physiol Rev 2014;94:355–82. 24692351 10.1152/physrev.00030.2013 PMC4044302

[9] Rohdenburg G Thyroid diabetes. Endocrinology 1920;4:63–70.

[10] Puri N Puri M Prevalence and pattern of thyroid disorders in diabetic patients. Ann Int Med Dental Res 2017;3:21–8.

[11] O’Meara N Blackman J Sturis J Alterations in the kinetics of C-peptide and insulin secretion in hyperthyroidism. J Clin Endocrinol Metab 1993;76:79–84. 8421108 10.1210/jcem.76.1.8421108

[12] Bech K Damsbo P Eldrup E β-Cell function and glucose and lipid oxidation in Graves’ disease. Clin Endocrinol 1996;44:59–66. 10.1046/j.1365-2265.1996.636458.x 8706294

[13] Levin R Smyth D The effect of the thyroid gland on intestinal absorption of hexoses. J Physiol 1963;169:755–69. 14103558 10.1113/jphysiol.1963.sp007294 PMC1368798

[14] Houssay BA The thyroid and diabetes. In: *Vitamins and Hormones*. Vol 4 Elsevier; 1946: 187–206.

[15] Brenta G Diabetes and thyroid disorders. Br J Diabetes Vasc Dis 2010;10:172–7.

[16] Rochon C Tauveron I Dejax C Response of glucose disposal to hyperinsulinaemia in human hypothyroidism and hyperthyroidism. Clin Sci 2003;104:7–15. 12519082 10.1042/

[17] Iwen KA Schröder E Brabant G Thyroid hormones and the metabolic syndrome. Eur Thyroid J 2013;2:83–92. 24783045 10.1159/000351249 PMC3821514

[18] Haggerty JL Reid RJ Freeman GK Continuity of care: a multidisciplinary review. BMJ 2003;327:1219–21. 14630762 10.1136/bmj.327.7425.1219 PMC274066

[19] O’connor PJ Desai J Rush WA Is having a regular provider of diabetes care related to intensity of care and glycemic control? J Fam Pract 1998;47:290–7. 9789515

[20] Ettner SL The relationship between continuity of care and the health behaviors of patients: does having a usual physician make a difference? Med Care 1999 547–55. 10386567 10.1097/00005650-199906000-00004

[21] Parchman ML Pugh JA Noël PH Continuity of care, self-management behaviors, and glucose control in patients with type 2 diabetes. Med Care 2002;40:137–44. 11802086 10.1097/00005650-200202000-00008

[22] Myers BA A Guide to Medical Care Administration: Concepts and Principles. Vol 1. New York: American Public Health Association; 1969.

[23] Knight JC Dowden JJ Worrall GJ Does higher continuity of family physician care reduce hospitalizations in elderly people with diabetes? Populat Health Manag 2009;12:81–6. 10.1089/pop.2008.0020 19361251

[24] Kim W Jang S-Y Lee T-H Association between continuity of care and subsequent hospitalization and mortality in patients with mood disorders: results from the Korea National Health Insurance cohort. PLoS One 2018;13:e0207740. 30452465 10.1371/journal.pone.0207740 PMC6242689

[25] Jang YJ Choy YS Nam CM The effect of continuity of care on the incidence of end-stage renal disease in patients with newly detected type 2 diabetic nephropathy: a retrospective cohort study. BMC Nephrol 2018;19:127. 29871604 10.1186/s12882-018-0932-3 PMC5989468

[26] Barker I Steventon A Deeny SR Association between continuity of care in general practice and hospital admissions for ambulatory care sensitive conditions: cross sectional study of routinely collected, person level data. BMJ 2017;356:j84. 28148478 10.1136/bmj.j84

[27] Hage M Zantout MS Azar ST Thyroid disorders and diabetes mellitus. J Thyroid Res 2011;doi: 10.4061/2011/439463. 10.4061/2011/439463 PMC3139205 21785689

[28] Wu P Thyroid disease and diabetes. Clin Diabetes 2000;18:38–138.

[29] Duntas LH Orgiazzi J Brabant G The interface between thyroid and diabetes mellitus. Clin Endocrinol 2011;75:1–9. 10.1111/j.1365-2265.2011.04029.x 21521298

[30] Mitrou P Raptis SA Dimitriadis GJER Insulin action in hyperthyroidism: a focus on muscle and adipose tissue. Endocr Rev 2010;31:663–79. 20519325 10.1210/er.2009-0046

[31] Dimitriadis G Baker B Marsh H Effect of thyroid hormone excess on action, secretion, and metabolism of insulin in humans. Am J Physiol 1985;248 (5 Pt 1):E593–601. 3887944 10.1152/ajpendo.1985.248.5.E593

[32] Chaker L Ligthart S Korevaar TI Thyroid function and risk of type 2 diabetes: a population-based prospective cohort study. BMC Med 2016;14:150. 27686165 10.1186/s12916-016-0693-4 PMC5043536

[33] Stanická S Vondra K Pelikánová T Insulin sensitivity and counter-regulatory hormones in hypothyroidism and during thyroid hormone replacement therapy. Clin Chem Lab Med 2005;43:715–20. 16207130 10.1515/CCLM.2005.121

[34] Park KH Lee EJ Recent review on medical treatment of thyroid disease. J Korean Med Assoc 2012;55:1207–14.

[35] Yi KH Moon JH Kim I-J The diagnosis and management of hyperthyroidism consensus—report of the Korean Thyroid Association. J Korean Thyroid Assoc 2013;6:1–1. 10.3803/EnM.2013.28.4.275 PMC3871036 24396691

[36] Cooper DS Propylthiouracil levels in hyperthyroid patients unresponsive to large doses: evidence of poor patient compliance. Ann Intern Med 1985;102:328–31. 2982303 10.7326/0003-4819-102-3-328

[37] Koopman RJ Mainous AG III Baker R Continuity of care and recognition of diabetes, hypertension, and hypercholesterolemia. Arch Intern Med 2003;163:1357–61. 12796073 10.1001/archinte.163.11.1357

[38] Gray DP Evans P Sweeney K Towards a theory of continuity of care. J R Soc Med 2003;96:160–6. 12668701 10.1258/jrsm.96.4.160 PMC539442

[39] Parchman ML Burge SK The patient-physician relationship, primary care attributes, and preventive services. Fam Med 2004;36:22–7. 14710325

[40] Singer PA Cooper DS Levy EG Treatment guidelines for patients with hyperthyroidism and hypothyroidism. JAMA 1995;273:808–12. 7532241

[41] Hamilton WB Integrating social support in nursing. J Adv Nurs 1994;20:1177–8.

[42] Ha KH Kim DJ Trends in the diabetes epidemic in Korea. Endocrinol Metab 2015;30:142–6. 10.3803/EnM.2015.30.2.142 PMC4508257 26194073

[43] Park B Suh S Sung J Improvement plan for validity of health insurance disease code and establishment of data application plan. Seoul National University College of Medicine, Health Insurance Review & Assessment Service; 2003.

